# Editorial: Stereotactic body radiotherapy for lung cancer

**DOI:** 10.3389/fonc.2023.1206736

**Published:** 2023-04-28

**Authors:** An Liu, Stephanie G. C. Kroeze

**Affiliations:** ^1^ Department of Radiation Oncology, City of Hope National Medical Center, Duarte, CA, United States; ^2^ Radiation Oncology Center KSA-KSB, Cantonal Hospital of Aarau, Aarau, Switzerland

**Keywords:** lung cancer, stereotactic radiation, immunotherapy, EGFR-inhibitors, early stage

Lung cancer remains the leading cause of cancer-related death worldwide, accounting for approximately 20% of all cancer deaths (1). Among the various treatment options for lung cancer, stereotactic body radiotherapy (SBRT) has emerged as an effective and minimally invasive treatment modality for early-stage non-small cell lung cancer (NSCLC) (2). SBRT delivers a high dose of radiation to the tumor while sparing the surrounding healthy tissue, resulting in high local control rates and excellent toxicity profiles (3).

With the advent of advanced imaging techniques and sophisticated radiation planning systems, SBRT can now be delivered to tumors in technically more challenging situations, such as bilateral or multiple lung lesions (4). Recently, its role is expanding beyond early-stage NSCLC and is increasingly used in combination with modern systemic treatments such as immunotherapy or EGFR-inhibitors in oligometastatic cancer patients (5).

In this special issue, several studies exploring the use of SBRT in more technically challenging situations are presented. The studies of Wang et al. and Eichkorn et al. elegantly evaluate SBRT in high-risk patients with locally advanced NSCLC. One of the most promising applications of SBRT in stage III lung cancer is the combination with immunotherapy to enhance the antitumor immune response (6). In their case series, Wang et al. reported 3 patients with stage III (N2) NSCLC who received neoadjuvant programmed cell death protein 1 (PD-1) blockade combined with SBRT before resection. The study showed that the combination therapy was well tolerated, with a high pathological response rate. The study suggests that neoadjuvant PD-1 blockade combined with SBRT could potentially improve the outcomes of patients with stage III non-small cell lung cancer (NSCLC) before surgery and is hypothesis-generating for future prospective trials.

A challenging situation for radiation treatment plans is when the primary tumor is located anatomically far away from the mediastinal lymph nodes. With conventionally fractionated volumetric arc therapy (VMAT), this often results in unnecessarily high lung radiation dose and it is known, that SBRT results in better outcome compared to conventionally fractionated radiotherapy of the primary tumor (7). The study by Eichkorn et al. investigated the use of SBRT in combination with VMAT for patients with locally advanced NSCLC where the primary tumor was peripherally located. The study included 21 high-risk patients who received SBRT to the peripheral primary lesion and VMAT to the mediastinal lymph nodes. This approach was well tolerated and resulted in an improved target volume coverage and significantly reduced dose exposure of the lungs, heart and spinal cord. The toxicity of this treatment concept was acceptable with no grade 4+ toxicities and to our opinion is an interesting option that should be explored in prospective trials.

The prognosis of metastatic lung cancer patients has improved drastically since the regular use of immunotherapy and targeted therapy. Nonetheless, especially with targeted therapy acquired resistance of the tumor is frequently observed. Since the early resistance is often limited, called oligoprogression, SBRT is now frequently applied to radically ablate the resistant clones with the goal to continue the targeted therapy. In this Research Topic, Chicas-Sett et al. set out to explore this concept in their mini review on metastatic NSCLC patients with EGFR-mutant NSCLC who developed resistance to epidermal growth factor receptor (EGFR) tyrosine kinase inhibitors and are subsequently treated with SBRT. This overview confirmed that the combination is a safe and effective approach that outweighs the risk of toxicity for the treatment of oligoprogressive NSCLC.

The efficacy of SBRT for simultaneous treatment of bilateral or multiple lung lesions is now also technically possible, but remains a challenge for treatment planning. One of the main objectives in treating bilateral lung lesions is to deliver a sufficiently high radiation dose to both lungs, while limiting the dose to the surrounding healthy tissue. In this issue, the study by Guo et al. compared the dosimetric and biological outcomes of single planning and double planning for bilateral lung cancer SBRT planning based on the Cyber-Knife system. The study found that double planning could lead to superior target coverage and conformity, while minimizing radiation exposure to healthy lung tissue and maintaining acceptable toxicity.

Another challenge in the use of SBRT for lung cancer is predicting treatment outcomes. In addition, the shape and volume of the primary tumor can impact the efficacy of SBRT treatment. The study by Duan et al. investigated the relationship between the gradient measure of lung SBRT treatment plans and the tumor volume and shape. They found that the gradient measure was significantly higher in tumors with irregular shapes and larger volumes, indicating that these tumors may be more challenging to treat with SBRT and could have a worse outcome. In the study by Yang et al., the authors investigated the relationship between CT appearance patterns after SBRT and treatment outcomes in early-stage NSCLC. They observed that patients with CT appearance patterns suggestive of fibrosis or consolidation had significantly better local control rates and progression-free survival compared to patients with patterns suggestive of tumor recurrence. The study suggests that the CT appearance pattern could be used to guide treatment decisions and predict outcomes in patients with early-stage NSCLC.

With the articles in this Research Topic, we hope to provide a review of the present role of and outcome of stereotactic radiotherapy for lung cancer treatment, including the technically more challenging situations of SBRT in stage III lung cancer, bilateral lung lesions or SBRT combined with immunotherapy or EGFR-inhibitors. As our understanding of the efficacy and limitations of SBRT continues to evolve, it is likely that this treatment modality will play an increasingly important role in the management of lung cancer in the coming years.

## Author contributions

SK and AL were guest editors for this topic. SK wrote the editorial, AL gave feedback. All authors contributed to the article and approved the submitted version.
